# Investigation of Antimicrobial Effects of Polydopamine-Based Composite Coatings

**DOI:** 10.3390/molecules28114258

**Published:** 2023-05-23

**Authors:** Rahila Batul, Mrinal Bhave, Aimin Yu

**Affiliations:** 1 Department of Chemistry and Biotechnology, School of Science, Computing & Engineering Technologies, Swinburne University of Technology, Hawthorn, VIC 3122, Australia; 2 Department of Pharmacology and Toxicology, College of Pharmacy, University of Ha’il, Ha’il 55211, Saudi Arabia

**Keywords:** polydopamine, gentamicin, silver nanoparticles, antimicrobial coatings

## Abstract

Herein, polydopamine (PDA)-based antimicrobial coatings loaded with silver nanoparticles (Ag NPs) and gentamicin were designed and prepared on glass slides using two different approaches. To our knowledge, this study was performed for the first time with the aim to compare these methods (viz., in situ loading and physical adsorption method) regarding the loading and release behavior of payloads. In one method, gentamicin was in situ loaded on PDA-coated substrates during PDA polymerization followed by Ag NPs immobilization (named as Ag@Gen/PDA); for the second method, Ag NPs and gentamicin were simultaneously loaded onto PDA via physical adsorption by immersing pre-formed PDA coatings into a mixed solution of Ag NPs and gentamicin (named as Ag/Gen@PDA). The loading and release characteristics of these antimicrobial coatings were compared, and both gave variable outcomes. The in situ loading method consequently provided a relatively slow release of loaded antimicrobials, i.e., approx. 46% for Ag@Gen/PDA as compared to 92% from physically adsorbed Ag/GenPDA in an immersion period of 30 days. A similar trend was observed for gentamicin release, i.e., ~0.006 µg/mL from Ag@Gen/PDA and 0.02 µg/mL from Ag/Gen@PDA each day. The slower antimicrobial release from Ag@Gen/PDA coatings would ultimately provide an effective long-term antimicrobial property as compared to Ag/Gen@PDA. Finally, the synergistic antimicrobial activities of these composite coatings were assessed against two microbial species, namely, *Staphylococcus aureus* and *Escherichia coli*, hence providing evidence in the prevention of bacterial colonization.

## 1. Introduction

Bacteria, despite being tiny creatures, have a threatening effect on human lives globally, owing to their infectious nature. Therefore, the development of efficient antibiotics has always been in high demand since the discovery of the first antibiotic, i.e., penicillin by Alexander Fleming [1]. Bacterial infection associated with medical implants and other biomedical devices is also a critical concern nowadays. Therefore, antimicrobial coatings are extremely required in various health care settings such as hospitals and industries to provide a sterile environment. In addition, due to the emergence of micro-organisms with multi-drug resistance capability, new developments became mandatory in the production of potent antimicrobial agents [2,3]. In this regard, the immobilization of silver nanoparticles (Ag NPs) in such clinical settings is of significance owing to their broad-spectrum antimicrobial and multidrug-resistant properties [4,5,6]. Ag NPs are believed to adhere to bacteria surfaces, cause DNA damage and alter membrane properties by passing through them. Moreover, Ag NPs can also impair microbial activities by releasing Ag^+^ ions. Hence, due to several mechanisms taking place at a time, bacteria cannot resist silver thus causing bacterial killing [7,8,9]. Ag NPs exhibit extensive antimicrobial properties and are used for treating infections, burns and chronic wounds. However, there are some limitations in terms of their particle aggregation, which cannot be ignored during practical usage, as these can diminish their antibacterial nature. Therefore, to overcome these deficiencies, Ag NPs have been tried to be immobilized and stabilized on many substrates such as silica, carbon nanotubes, iron oxide or carbonaceous nanomaterials [10,11]. To achieve their successful stabilization, the processes need to pass through a series of complex procedures, including the use of surfactants and reducing agents. In addition, these processes can lead to the usage of toxic reductants, hence causing environmental toxicity and biohazards [12,13,14]. Therefore, for this, green synthetic procedures are required that are cost-effective, environmentally friendly, non-toxic and lead to desired biocompatibility and antimicrobial efficacy.

Polydopamine (PDA), a mimic of adhesive proteins found in mussels, has been investigated in various biomedical fields such as biotechnology, bone and tissue engineering, drug delivery and chemo- and photothermal therapies [15,16,17,18]. Owing to its bio-compatible, biodegradable and non-toxic nature, there are few concerns with its implementation in various biomedical fields [19,20]. The adhesive and reductive properties of PDA offer the attachment and uniform distribution of Ag NPs on various PDA-coated substrates reported so far [21,22,23,24]. The Ag^+^ ions could likely be absorbed due to their metal-binding capabilities of phenolic hydroxyl groups followed by their reduction into Ag NPs by catechol groups of PDA. Furthermore, the PDA layer could act as a “bio-glue” for the stabilization and immobilization of Ag NPs on its surface [25,26]. For example, Ag@PDA could be used as a surface-enhanced Raman scattering (SERS) substrate for the detection of myocardial infarction via analyzing troponin levels [27]. Silver-composited PDA NPs hydrogels obtained by a similar green synthetic procedure were reported recently for antibacterial purposes [28]. Likewise, dopamine-assisted deposition of Ag NPs on hydroxyethyl cellulose was also reported to be used as catalytic material [29].

Previous research proposed many different types of mechanisms of bactericidal activity associated with Ag NPs. These involve the generation of reactive oxygen species, free radicals generated from the surface of Ag NPs, released Ag^+^ ions stress, as well as other interactions with bacterial cell walls via forming linkages with the respiratory enzymes or depleting the levels of intracellular ATP [30,31]. The exact modes of action of the bacteriostatic interaction of Ag NPs remain under investigation, as to whether Ag NPs or the released Ag^+^ causes bacterial inhibition. However, most of the studies are in favor of Ag^+^ for the antibacterial activity, while Ag NPs may or may not directly induce toxicity. 

The synergistic properties of Ag NPs in a combination of antibiotics such as tetracycline and neomycin have also been investigated [32]. These reported antimicrobial combinations showed successful synergistic inhibitory impacts on the growth of multi-drug-resistant bacteria. Some studies have also proven the inhibition of biofilm formation by using Ag NPs with other antibiotics [33]. This is because of the generation of hydroxyl radicals by Ag NPs to enhance bactericidal effects. Moreover, bactericidal behavior is also believed to occur due to the formation of Ag NPs and antibiotic complexes [34]. Gentamicin being an aminoglycoside antibiotic is broadly used against a variety of bacterial infections. It has also been proven to show strong synergistic abilities with Ag NPs to kill bacteria and inhibit biofilm formation [35,36]. Ag NPs are responsible for the inhibition of bacterial invasion by rupturing their membranes, whereas gentamicin works by irreversibly binding to the 30S subunit of the bacterial ribosome, hence interrupting protein synthesis [32,37]. Moreover, the synergistic antimicrobial phenomenon of Ag NPs in combination with PDA has also been evaluated to possess against *Escherichia coli,* suggesting that catechol linkage between Ag and PDA and hydroxyl groups of PDA are mainly responsible for governing antimicrobial activities [38].

This study focuses on the production of antimicrobial PDA coatings by attempting a combinatorial approach. A PDA coating is applied as support for the immobilization of silver NPs as well as the loading of gentamicin via hydrogen bonding and electrostatic interactions between the hydroxyl group of the PDA and the amine group of gentamicin [39,40]. Our group previously reported in situ method for gentamicin loading in PDA NPs, which provided outstanding results in terms of the loading and release behavior of the payloads [39,40]. Following that, herein, the in situ loading method has been reported for loading antimicrobials on PDA coatings. Moreover, comparable studies have also been performed for the first time between two methods, viz., in situ loading and physical adsorption method for loading antimicrobials in terms of their loading and release behaviors. For the first method, gentamicin is in situ loaded with PDA on the glass slide, followed by the immobilization of Ag NPs, whereas in the second method, gentamicin and Ag NPs are post-loaded on pre-formed PDA-coated glass slides. Furthermore, the loading and release behavior of Ag NPs and gentamicin were compared in each method, as well as the antimicrobial activities of resulting coatings. Long-term silver and gentamicin release are carried out for at least 30 days, and quantification is carried out by inductively coupled plasma-atomic emission spectroscopy (ICP-AES) and liquid chromatography–mass spectrometry (LC-MS), respectively. The determination of their release profile is critical for understanding their long-term antibacterial effects. Therefore, their synergistic antimicrobial properties have also been investigated by various techniques.

## 2. Materials and Methods

### 2.1. Material

Dopamine hydrochloride, ammonia aqueous solution (NH_3_ content: 38–40%) and ethanol were purchased from Sigma-Aldrich Pty. Ltd., Sydney, NSW, Australia). Gentamicin sulfate (active fraction: 590 μg gentamicin/mg) was obtained from AK Scientific (Union City, CA, USA). Silver nitrate was purchased from Merck Pty. Ltd., (Darmstadt, Germany). 

Piranha solution was prepared from three parts concentrated sulfuric acid and one part 30% hydrogen peroxide. Tris buffer (10 mM; pH 8.5) was prepared by dissolving Tris base and pH was adjusted with 1 M hydrochloric acid (HCl). The chemicals used in all experiments were of analytical grade and applied without further purification. Microscope glass slides were cut into approximately 1 × 1 cm^2^ by using a diamond pen, washed with piranha solution followed by drying prior to use in experiments. The Milli Q water (fitted with a Millipak^®^ 40 filter unit) with a resistivity of 18.2 MΩ cm was used in all experiments. 

Nutrient agar and nutrient broth were supplied by Acumedia, Melbourne Australia. The microbial species *Staphylococcus aureus* (ATCC 25923) and *Escherichia coli* (ATCC 25922) were obtained from Swinburne University of Technology microbial collection.

### 2.2. Methodology

#### 2.2.1. Preparation of PDA-Coated Glass Slides (PDA@glass)

Glass slides were washed thoroughly with freshly prepared piranha solution. Slides were then incubated in Tris buffer (pH 8.5) containing DA (2 mg/mL) at different time intervals of 18, 20, 22 and 24 h, and then sonicated for 10 min in water to remove large aggregates and dried in the open air at room temperature. 

#### 2.2.2. Deposition of Silver NPs on PDA Coatings (Ag@PDA)

**Figure 1:**
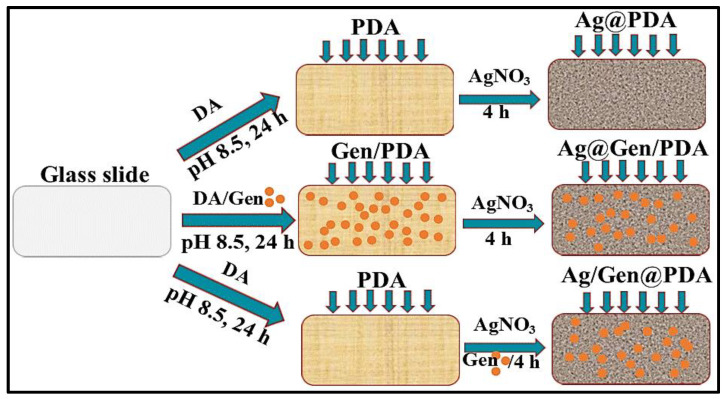
Schematic procedures of preparing Ag NP- and gentamicin-loaded PDA coatings.

Silver NPs were deposited on PDA-coated glass slides by the one-step reduction method. Briefly, PDA-coated glass slides were incubated in 50 mM AgNO_3_ water solution for 4 h. The slides were then rinsed with water and dried in the air. These were named Ag@PDA, as shown schematically in Figure 1.

#### 2.2.3. Deposition of Gentamicin and Ag NPs on PDA Coatings (Ag/Gen@PDA)

A mixed solution of 1 mg/mL of gentamicin and 50 mM of AgNO_3_ in water was prepared by vigorous stirring for at least 15 min. A few drops of aqueous ammonia solution (28–30%) were added to the above mixture until the solution became clear. Then, PDA-coated glass slides were dipped in that solution for 4 h, followed by rinsing several times with water and drying. These slides were named Ag/Gen@PDA as shown schematically in Figure 1.

#### 2.2.4. Deposition of Silver on Gentamicin Loaded PDA Coatings (Ag@Gen/PDA)

A solution of DA (2 mg/mL) and gentamicin (1 mg/mL) was prepared in Tris buffer. The glass slides were dipped in it for 24 h to produce gentamicin-loaded PDA coatings. Then, these glass slides were slightly rinsed with water and left for drying. These slides were named Gen/PDA-coated glass slides. The as-prepared Gen/PDA-coated glass slides were then dipped in AgNO_3_ solution for 4 h to allow silver deposition, followed by rinsing and drying steps. These glass slides were named Ag@Gen/PDA as schematically shown in Figure 1.

### 2.3. Characterization of PDA Coatings 

FTIR spectra were obtained using the Nicolet iS5 model FTIR (Thermo Fisher Scientific Company, Waltham, MA, USA) to investigate the characteristic functional groups on PDA-coated glass slides. The surface morphology and elemental compositions of silver de-posited PDA-coated substrates were determined by a field emission Scanning Electron Microscope (FeSEM, ZEISS SUPRA 40VP, Jena, Germany) at an acceleration voltage of 3 kV, which was equipped with energy-dispersive X-ray spectrometry (EDX). The UV-Vis absorption spectra were recorded by using the HALO RB-10 UV-Vis Ratio Beam spectrophotometer (Scientific Pty. Ltd., Victoria, Australia). PDA-coated quartz slides were hung with sticky tape across the UV-light pathway for the determination of successful PDA coating.

### 2.4. Quantification of Silver Release from Glass Slides

To investigate the stability and long-term silver release behavior, the coated glass slides were immersed in 5 mL of water under static conditions for at least 30 days. After every 24 h, the leaching medium was collected, followed by replacement with fresh water. The leaching media were acidified by adding a few drops of 98% HNO_3_ prior to determining the concentration of Ag^+^ ions by using inductively-coupled plasma atomic emission spectrometry (ICP-AES) (Thermo scientific iCAP 7000 series, Cambridge, UK).

### 2.5. Quantification of Gentamicin Release from Glass Slides

Ag/Gen@PDA- and Ag@Gen/PDA-coated glass slides were immersed in 2 mL water under the same conditions as used for silver release quantification described in the above section. The drug release medium was collected every day and replaced by 2 mL of fresh water. The concentration of gentamicin release was determined using LC-MS under the same column conditions. Briefly, a 20 µL injection volume was used, and the concentration was obtained by using the calibration curve from 0.1 to 0.5 µg/mL.

### 2.6. Antimicrobial Experiments for Glass Slides

To determine the synergistic antibacterial activity of antimicrobial-coated glass slides, three different methods were performed. Two bacterial species were chosen as models to assess their antimicrobial properties, namely, a Gram-positive *Staphylococcus aureus* (ATCC 25923) and Gram-negative *Escherichia coli* (ATCC 25922). PDA-coated glass slides were used as a control.

### 2.7. Zone of Inhibition (ZOI) 

The method of the zone of inhibition was performed using a modified protocol of the CLSI MO7-A9 method [41]. Briefly, aliquots of 100 µL of standard bacterial culture, viz., 105 and 103 CFU/mL from each bacterial strain (*S. aureus* and *E. coli*) were spread onto nutrient agar plates. The UV-sterilized antimicrobial coatings were then placed onto the plates. After incubation at optimal temperature (37 °C), for 24 h, zones of inhibition formed around each glass slide were measured by using a Vernier caliper. Measurements were taken diagonally as well as perpendicularly, and then average measurements were calculated to document the average zone of inhibition. The plates were prepared in duplicates. 

### 2.8. Measurements of Optical Densities (OD600)

All the glass slides were immersed in the standard bacterial inoculum (105 and 108 CFU/mL for *S. aureus* and *E. coli*, respectively) in 12-well flat bottom plates. The plates were then incubated for 24 h at 37 °C. In all cases, bacterial growth in the broth medium was evaluated by optical density (OD) at 600 nm with a Thermo Scientific Helios™ Epsilon Visible spectrophotometer (Victoria/Australia). The absorbance of nutrient broth was reduced to zero before measuring the optical densities of incubation. Duplicate samples were tested.

### 2.9. Spread Plate Method

The number of viable bacteria was quantified by using the spread plate method [42] with minor modifications. Briefly, each glass slide was immersed in 2 mL of 105 and 108 CFU/mL for *S. aureus* and *E. coli*, respectively, for 24 h. After vortexing the reacted broth media for 30 s, 100 µL aliquots were spread on nutrient agar plates. Duplicate plates were prepared and incubated for another 24 h at 37 °C. Bacterial growth was observed, and images were captured by using a digital camera for evidence. 

## 3. Results and Discussion

### 3.1. Preparation and Characterization of PDA-Based Antibacterial Coatings

To demonstrate the use of PDA as a substrate for incorporating various antibacterial agents, we produced several coatings composed of Ag NPs and gentamicin using different procedures. Firstly, glass slides were coated with PDA by the self-polymerization method at various time intervals from 18 to 24 h. Then, Ag NPs and gentamicin were loaded onto PDA coatings either via in situ polymerization along with PDA, or by using the post-loading method. In the method that gentamicin was in situ loaded with PDA on the glass slide, followed by the immobilization of Ag NPs, these coatings were named as Ag@Gen/PDA. In the other method, gentamicin and Ag NPs were post-loaded on PDA-coated glass slides, and these coatings were named as Ag/Gen@PDA. The procedures are schematically shown in Figure 1. These approaches were intended to determine the impact of different loading strategies on the release profile and antimicrobial performance of resulting coatings.

**Figure 2:**
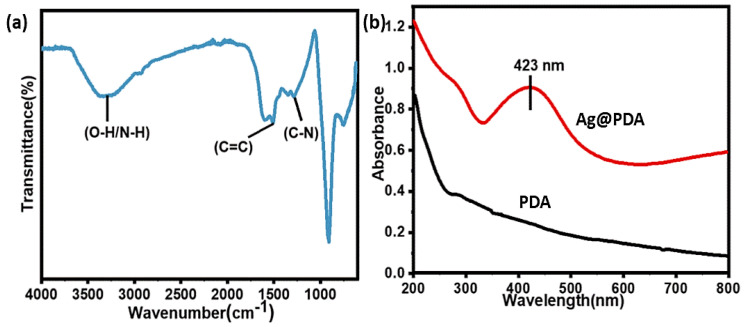
(**a**) FTIR spectrum of PDA coating, (**b**) UV–Visible spectra of PDA- and Ag@PDA-coated quartz slide.

PDA was successfully coated on glass and quartz slides at different time intervals, which was revealed by various characterization techniques. For example, FTIR spectra demonstrated characteristic absorption peaks at 1507 cm^–1^, indicating the C=C of the indole structure of PDA. The broader peak at 3328 cm^–1^ corresponds to N-H and O-H stretching and the smaller peak at 1284 cm^–1^ was attributed to C-N stretch in indolequinone [43] as shown in Figure 2a. 

**Figure 3:**
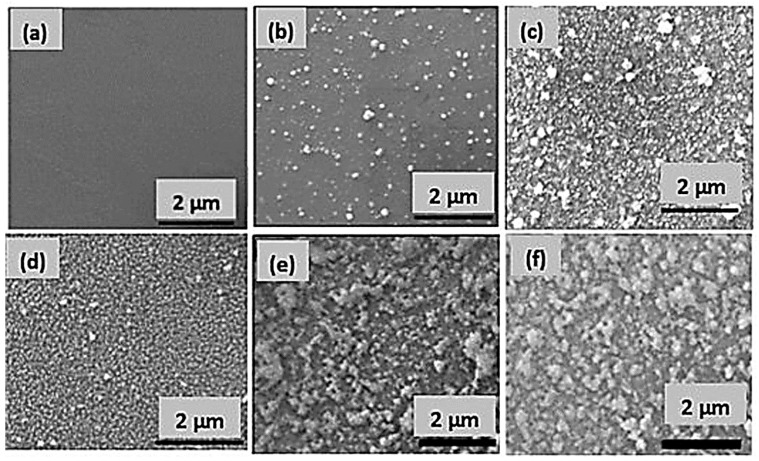
(**a**) SEM image of uncoated, (**b**) PDA, (**c**) Ag@PDA, (**d**) Ag/Gen@PDA, (**e**) Gen/PDA and (**f**) Ag@Gen/PDA-coated glass slides.

Furthermore, UV–Visible spectrophotometry also confirmed coatings on quartz slides. It was observed that the absorbance of the PDA-coated slide increased with increasing polymerization time from 18 to 24 h, which can indirectly indicate the increment in coating thickness with respect to time, as shown in Figure S1a. By considering the absorbance around 400 nm [44], a calibration curve was drawn which also showed a good linear relationship with respect to time with a reliable R-square value of 0.9945, as shown in Figure S1b. Hence, the deposition of PDA seems proportional to the polymerization time of DA, which provides an easy method to effectively control the thickness of the PDA coating on the flat substrates. A visual observation also showed the change in the color of the glass substrate from colorless to dark brown after PDA coating, as shown in Figure S2. Moreover, an SEM image of the PDA-coated glass slides also proved the successful PDA coating by observing a remarkable morphology difference between the uncoated and PDA-coated glass slides as shown in Figure 3a,b.

### 3.2. Deposition of Silver NPs on PDA (Ag@PDA) Coatings

Further, Ag NPs were deposited on PDA-coated substrates and named as Ag@PDA. It was observed that after immersion in AgNO_3_ solution for 4 h, the color of the PDA substrate turned silvery grey, indicating the generation of Ag NPs, as shown in Figure S2. Similarly, UV–Visible spectroscopy analysis was also carried out to confirm the silver deposition. The formation of Ag NPs was evidenced by an increase in the absorbance between 380 and 450 nm [45] as shown in Figure 2b. 

**Figure 4:**
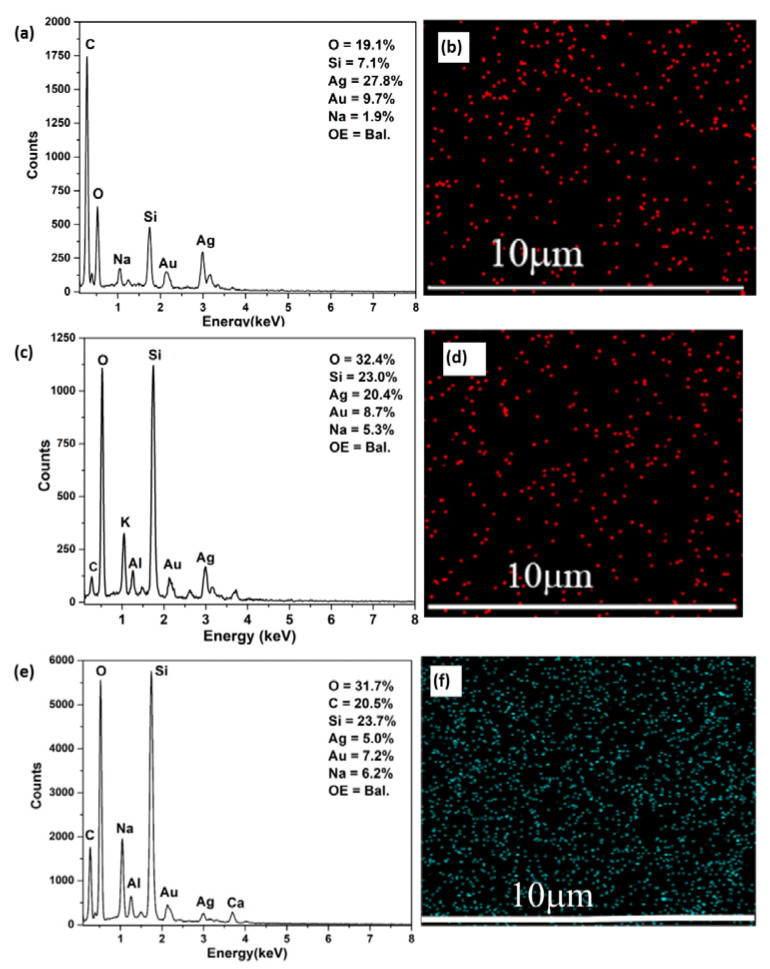
(**a**) EDX spectrum and (**b**) elemental mapping of Ag@PDA; (**c**) EDX spectrum and (**d**) elemental mapping of Ag/Gen@PDA; (**e**) EDX spectrum; and (**f**) elemental mapping of Ag@Gen/PDA-coated glass slides. OE in (**a**,**c**,**e**) representing other elements.

To further confirm the presence of surface-bound Ag NPs, SEM and EDX analyses were carried out. SEM image confirmed the deposition of Ag NPs on PDA-coated substrates, as shown in Figure 3c. EDX mapping analysis revealed the elemental content and their locations on the PDA-coated substrate (Figure 4a,b). The major components were found to be carbon, which originated from the PDA and the Ag atoms of the Ag NPs (red spots in Figure 4b). These results further indicate that the Ag NPs were uniformly distributed on the surface, most likely due to the presence of the catechol groups on PDA coating.

The versatile chemistry of PDA can provide the surface metallization of a wide range of substrates simply and effectively. Therefore, the reductive capacity of PDA enables the deposition of metallic films when coming in contact with noble metal salt solutions. Thereby, silver deposition is mostly attributed to redox reactions between catechol groups on PDA coatings and Ag^+^, which results in the formation of Ag^0^ at its surface. Notably, the synthetic mechanism of PDA remains elusive; however, it has been proposed that dopamine undergoes self-polymerization and cross-linking, leading to the formation of PDA. Further, the catechol moiety of PDA chelates with Ag^+^ ions, and then Ag^+^ can be in situ reduced to Ag NPs. Meanwhile, the catechol group oxidizes to quinone leading to the formation of PDA [42]. The proposed mechanism of the chelation and reduction of Ag ions can be seen in Figure S3.

Furthermore, dual antibacterial coatings such as Ag/Gen@PDA and Ag@Gen/PDA were also characterized. Visual photographs were obtained (as shown in Figure S2) to determine the morphological changes after the sequential deposition of silver and gentamicin on such substrates. Clear differences for each coating can be seen, which can be further revealed by SEM analysis. Figure 3d shows the uniform deposition of Ag and Gen on PDA-coated glass slides on Ag/Gen@PDA. EDX and elemental mapping, as shown in Figure 4c,d, confirmed the distribution of silver atoms on the PDA coatings; however, for the determination of gentamicin loading, further characterization was required. Moreover, for Ag@Gen/PDA glass slides, gentamicin was firstly in situ loaded with PDA during the polymerization reaction. This time, the sonication of PDA-coated glass slides was avoided to prevent the release of gentamicin from the coatings. Then, further modification was carried out by depositing Ag NPs on the Gen/PDA coating to develop a Ag and gentamicin dual-loaded PDA coating (i.e., Ag@Gen/PDA). SEM analysis (Figure 3e,f) does not show any morphological change before and after silver deposition. For this, EDX and elemental mapping were carried out to confirm the presence of Ag NPs, as shown in Figure 4e,f. Overall, based on SEM images, Ag/Gen@PDA coatings were found to be smooth with uniformly distributed Ag NPs, and fewer PDA aggregates were observed. Moreover, EDX analysis and elemental mapping showed a large number of silver atoms on the surface. On the other hand, Ag@Gen/PDA glass coatings showed aggregated PDA, which might be because of a reduction in sonication time to avoid gentamicin loss from the surface. Furthermore, elemental mapping from EDX analysis showed lower amounts of silver atoms (Figure 4f). This might be because most of the PDA catechol moieties could be utilized by gentamicin, leaving less for the reduction of silver ions.

### 3.3. Quantification of Sliver Loading and Release from PDA Coatings

The long-term stability and Ag^+^ release profile are essential factors for exhibiting long-term antibacterial effects, which can only be achieved by the gradual release of silver ions from coatings. To determine the silver release, silver immobilized substrates were immersed in water, and the leaching medium was collected at predetermined time intervals followed by medium refreshing. This process was continued for at least 30 days. The leaching medium was acidified by adding a couple of drops of 98% nitric acid to form silver ions. The silver content was then quantified by ICP-AES. Silver nitrate solutions with a series of concentrations were prepared as standard to reach the final Ag+ concentrations from 0.1 to 1.5 µg/mL to obtain a calibration curve. The non-cumulative and cumulative release profiles were obtained for each of the coatings, viz., Ag@PDA, Ag@Gen/PDA- and Ag/Gen@PDA. After the completion of 30 days’ immersion and the collection of leaching media, the glass slides were immersed in 3.5% nitric acid to quantify the remaining amount of loaded silver on the substrates. The obtained values were summed up with the released Ag^+^ concentrations obtained for each coating to indirectly determine the unknown concentration of silver that had already been loaded onto the substrates.

#### 3.3.1. Quantification of Silver Release from Ag@PDA

The detachment of silver in the form of Ag^+^ ions is essential for long-term antimicrobial effects and biocompatibility. Therefore, long-term silver release behavior was required, and data were analyzed by plotting both non-cumulative and cumulative release curves. The release profile for Ag@PDA was studied as a control.

**Figure 5:**
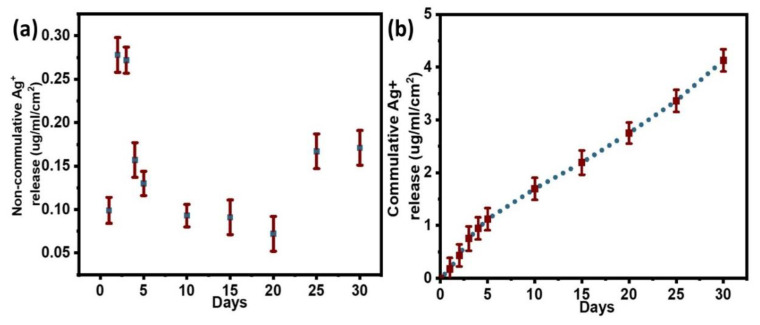
(**a**) Non-cumulative and (**b**) cumulative silver release curves obtained from Ag@PDA.

As shown in the non-cumulative release curve in Figure 5a, the initial release amount occupied a small fraction of total loaded Ag^+^, i.e., approximately 0.1 µg/mL, followed by a gradual increase for up to the next 2 days. After that, the sustained slow release was observed over an extended period (up to twenty days), and then the release rate was faster again due to unknown reasons. Overall, a consistent Ag^+^ release with an average rate of 0.1 to 0.2 µg/mL/day could comply with the long-term anti-microbial effects of substrates. The cumulative release curve in Figure 5b shows overall clear linearity for Ag^+^ release, indicating a sustained slow release even after 30 days. By the end of the immersion test, the cumulative Ag^+^ concentration was approximately 3.9 µg/mL, which is approximately 39.5% of the total loaded silver. The remaining amount of silver shows good evidence of its long-term antibacterial effects.

#### 3.3.2. Quantification of Silver Release from Ag/Gen@PDA

**Figure 6:**
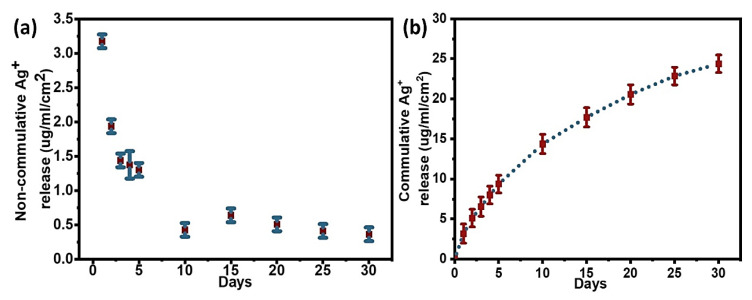
(**a**) Non-cumulative and (**b**) cumulative silver release profile for Ag/Gen@PDA.

The Ag/Gen@PDA coating was prepared by loading silver ions and gentamicin on PDA-coated glass slides. Ag has a high affinity for nitrogen; we thus believe silver ions were adsorbed at nitrogen sites of PDA and then reduced by catechol functional groups of PDA. As mentioned before, gentamicin could be loaded by electrostatic interaction and hydrogen bonding with PDA. Moreover, Ag NPs could also form chemical bonds with the nitrogen of amino groups of gentamicin, leading to the formation of a Ag/Gen complex as hypothesized in literature [32]. Therefore, a higher amount of silver loading was observed on Ag/Gen@PDA coatings, i.e., 26.44 µg/mL/cm^2^, which is approximately twice as compared to Ag@PDA coatings. In the former, Ag^+^ release was significantly higher than Ag@PDA, which is obvious due to the higher concentration gradient. Therefore, the silver release was abrupt initially, with approximately 3.1 µg/mL/cm^2^ on the first day followed by a gradual decrease for the next 6 days. Then, the release rate was sustained to an average of 0.5 µg/mL/cm^2^/day for the rest of the 30 days, as shown in the non-cumulative silver release curve in Figure 6a. Moreover, the cumulative release curve (Figure 6b) shows an almost similar release trend to Ag@PDA. However, a higher percentage of silver was lost in a similar duration of 30 days. 

#### 3.3.3. Quantification of Silver Release from Ag@Gen/PDA

**Figure 7:**
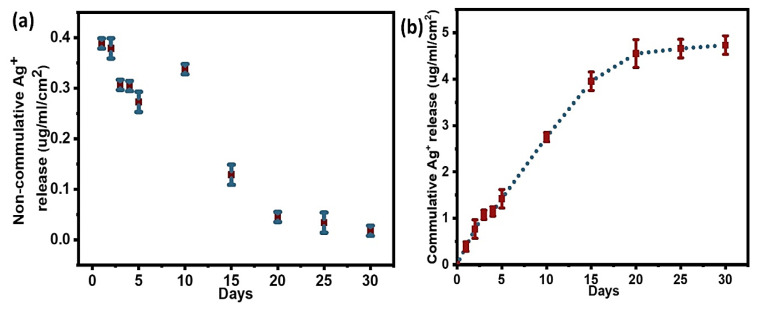
(**a**) Non-cumulative and (**b**) cumulative silver release profile for Ag@Gen/PDA.

In contrast to the above, for the Ag@Gen/PDA coatings, after experiencing a burst release of Ag^+^ on the first day, i.e., 0.4 µg/mL, a gradual decrease was observed over the next few days. After that, an increase in rate was observed again on the tenth day of immersion due to unknown reasons, followed by a gradual decrease in the release rate for the rest of the 30 days as shown in Figure 7a. However, the cumulative Ag^+^ release profile (Figure 7b) shows a very similar trend to Ag@PDA and Ag/Gen@PDA. The total amount of silver loading was almost similar to Ag@PDA. However, Ag^+^ seemed to be released at a higher rate than Ag@PDA. It might be attributed to the masking of some of the active sites of PDA by gentamicin, as it has been in situ loaded with PDA. Therefore, due to the non-availability of catechol functional groups of PDA, Ag NPs could only form complexes with hydroxyl groups of gentamicin, which could detach Ag^+^ easily. Recent studies have also suggested a higher release rate of Ag^+^ due to the formation of antibiotic and Ag NP complexes as compared to Ag@PDA. [32,46]. Overall, at the end of the immersion test, only 45.8% of the total silver was released, hence showing a long-term release profile.

In conclusion, combined analytical studies show some merits and also some shortcomings of as-prepared coatings. The in situ loading of Ag and gentamicin upon PDA coating, referred to as Ag/Gen@PDA-coated glass slide, is capable of loading higher amounts of Ag as compared to Ag@Gen/PDA coating. As a consequence, a large percentage of silver (approximately 92.2%) released in only 30 days of time might lead to toxic effects of silver, as shown in Table 1. On the contrary, Ag@Gen/PDA coating with a slower release profile could reach approximately 46% of the total loaded silver (Table 1). Hence, the consistent release of silver ions through this process could imply promising long-term antibacterial ability due to the slower diffusion of Ag^+^ ions. 

### 3.4. Quantification of Gentamicin Release from PDA Coatings

The quantification of gentamicin release from the as-prepared PDA coatings was carried out by LC-MS. Here, we kept a 20 µL injection volume, because only a very small amount of gentamicin will release from approximately 1 cm^2^ PDA coating. Unlike silver loading quantification, it was not possible to carry out the quantification of the gentamicin loading amount, because there might be the formation of gentamicin-loaded PDA NPs in the reaction media. Therefore, it was impossible to remove those gentamicin-loaded NPs during in situ loading, as this could ultimately give a false detection of unloaded gentamicin in supernatants. 

**Figure 8:**
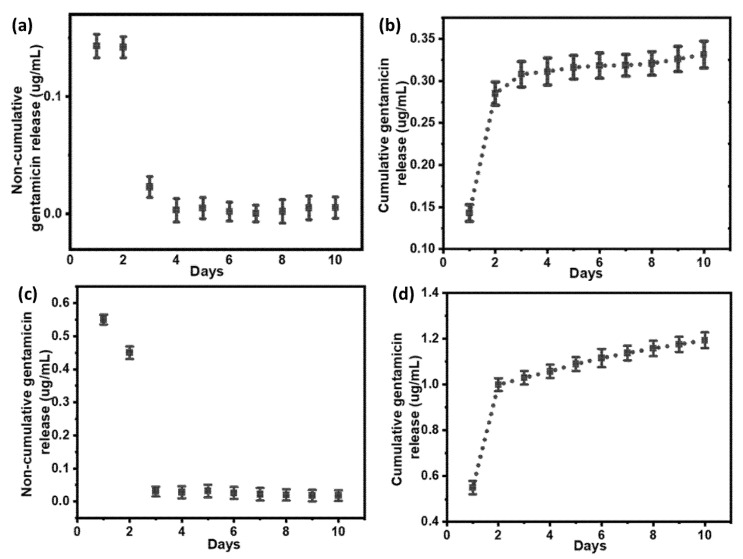
(**a**) Non-cumulative and (**b**) cumulative gentamicin release from Ag@Gen/PDA-coated glass slides. (**c**) Non-cumulative and (**d**) cumulative gentamicin release from Ag/Gen@PDA-coated glass slides.

Initially, the premature release of gentamicin was observed from both Ag@Gen/PDA and Ag/Gen@PDA coatings in the first few days. It was attributed to the concentration gradient difference between the coating and the released medium. From day 3 to day 10, a very small amount of gentamicin (~0.006 µg/mL from Ag@Gen/PDA and 0.02 µg/mL from Ag/Gen@PDA) was released each day as shown in the non-cumulative gentamicin release data in Figure 8a,c. It is noted that gentamicin could only be detected for up to 10 days. After 10 days, the released gentamicin was too little to be detected. Hence, further data could not be quantified.

From the gentamicin release data, it was possible to compare the two different loading approaches. In the Ag@Gen/PDA coating, less total gentamicin, i.e., approximately 0.392 µg/mL/cm^2^ (Figure 8b), was released in the first 10 days as compared to Ag/Gen@PDA, from which approximately 1.19 µg/mL/cm^2^ (Figure 8d) was released. This observation is very similar to silver release data. It might also be for the same reasons, as gentamicin was in situ loaded in Ag@Gen/PDA, hence forming hydrogen bonds and electrostatic interactions with PDA. Therefore, a very slow and sustained release was observed. This observation could also be compared to gentamicin release from G-PDA NPs in our published work [40], where the in situ loading method was used for its loading, consequently giving a slow and long-term release. On the other hand, a higher concentration of gentamicin release was observed in Ag/Gen@PDA, where gentamicin was physically adsorbed using the post-loading method, which might be loosely attached and gave release at a faster rate. 

Hence, it can be concluded that the in situ loading method can achieve long-term release and ultimately long-term antibacterial effects than the post-loading method and could be developed further. 

### 3.5. Evaluation of Synergistic Antimicrobial Properties of Silver and Gentamicin Loaded PDA Coatings

The release of silver and antibiotics was monitored and analyzed for at least 30 days, which is very important to determine the antimicrobial effects of PDA coatings. To further evaluate the potential synergistic effects of the loaded silver and gentamicin, a series of antimicrobial activity testing experiments were designed and conducted to evaluate the performance of various PDA coatings on inhibiting bacterial growth. 

#### 3.5.1. Zone of Inhibition

The antibacterial-coated glass slides were incubated in nutrient agar plates with *S. aureus* (105 CFU/mL) or *E. coli* (103 CFU/mL) for 24 h. Clear zones of inhibition could be seen against *S. aureus* and *E. coli* (Figures S4 and S5, respectively). Bacterial growth could be observed on the PDA-coated glass slide, which gives a clear indication that PDA itself does not possess antimicrobial activity. On the contrary, all other glass slides (Ag@PDA, Gen/PDA, Ag@Gen/PDA and Ag/Gen@PDA) showed clear inhibition zones. Table 2 summarizes the measured zones of inhibition formed around each slide.

From the measurements, it could be concluded that Ag@PDA and Gen/PDA that possess either Ag NPs or gentamicin could effectively prevent bacterial growth. This is probably caused by the diffusion and release of silver or gentamicin in their vicinity. Additionally, glass slides with both silver and gentamicin, i.e., Ag@Gen/PDA and Ag/Gen@PDA, show significantly larger inhibition zones as compared to Ag@PDA and Gen/PDA slides, strongly suggesting the phenomenon of synergism. Moreover, Ag/Gen@PDA showed larger zones of inhibition as compared to Ag@Gen/PDA, hence proving the faster release rate of silver and gentamicin from post-loaded coatings.

#### 3.5.2. Optical Density Measurements

The antibacterial activity of antibacterial-coated glass slides was also determined in nutrient broth solution against the chosen microbial species as shown in Figure S6. Glass slides were incubated in each of the bacterial standard suspension *S. aureus* and *E. coli* adjusted to 105 and 108 CFU/mL, respectively for 24 h. Solution turbidity was observed visually, and the optical densities at 600 nm were measured to assess the antimicrobial activities as shown in Table 3. Visible bacterial growth was observed with uncoated and PDA-coated glass slides. Therefore, these two slides were referred to as controls showing obvious turbidity as well as higher OD values. The nutrient broth solution alone was reduced to zero, before measuring OD values for controls (uncoated glass slides) as well as samples.

Ag@PDA and Gen/PDA showed comparatively less turbidity in the media, supported by a massive decrease in their optical density, showing that Ag@PDA and Gen/PDA possessed strong activity against *S. aureus* and *E. coli*. Moreover, nutrient broth solutions with dual-coated silver and gentamicin glass slides, i.e., Ag@Gen/PDA and Ag/Gen@PDA, remained very clear or least turbid, suggesting a significant decrease in bacterial reduction. Here, a difference was observed between the two different synthetic methods, i.e., Ag/Gen@PDA showed 10 times less turbid solution and lower optical density values as compared to Ag@Gen/PDA, in accordance with the drug release data and method of loading. However, their synergetic antimicrobial activities are confirmed by a decrease in OD values.

#### 3.5.3. Spread Plate Method

The as-prepared glass slides were incubated in the standard bacterial suspensions (105 and 108 CFU/mL for Gram-positive, *S. aureus* and Gram-negative, *E. coli*, respectively) for 24 h. Then, 100 µL of the bacterial suspension of the respective CFU was uniformly coated on nutrient agar plates by a spreader. After incubation for 24 h at 37 °C, bacterial growth was observed on the plates. Figure S7a,b show the antimicrobial activity against both *S. aureus* and *E. coli* after a 24 h incubation period. A large number of colonies were observed after treatment with Ag@PDA and Gen/PDA, showing that silver and gentamicin when coated on glass slides alone are unable to inhibit bacterial growth independently. In contrast, after treatment with Ag/Gen@PDA and Ag@Gen/PDA, no colony formation was observed on agar plates, confirming the growth inhibition of both bacteria. Their combined antimicrobial effects are visible in Ag/Gen@PDA and Ag@Gen/PDA agar plates and comply with the synergetic effect.

Previous studies have proposed several antimicrobial mechanisms for Ag NPs. It has been suggested that Ag NPs could attack the respiratory chain of different microorganisms, by reacting with oxygen, ultimately leading to cell death [47]. Moreover, Ag NPs also cause the inhibition of the unwinding of DNA, hence interrupting different cellular processes [48]. Ag NPs are toxic, if used in higher concentrations, therefore, it is recommended that they are used in lower concentrations in combination with antibiotics to facilitate their antibacterial activity [49]. 

The synergistic action mechanism of Ag NPs and gentamicin is still under investigation. Several studies reported that, as compared to Ag NPs alone, the combination of antibiotics and Ag NPs could cause Ag^+^ to be released at a higher rate [32,46]. Our study on silver release from Ag/Gen@PDA and Ag@Gen/PDA also supported this finding, as shown in Figure 6 and Figure 7. It has also been proposed that the combination of antibiotics with Ag NPs through the active groups of antibiotics such as hydroxyl and amino groups results in the formation of conjugates. The synergistic effect may be due to the delivery of antibiotic–Ag NP conjugates to the cell. Gentamicin is hydrophilic, whereas Ag NPs are hydrophobic. There is a hydrophobic section in cellular membranes [50]. Thereby, Ag NPs and their conjugates can pass through the cellular membrane to be delivered to the interior of bacterial cells [51,52]. The combination is frequently used, especially when the bacteria become resistant [53].

## 4. Conclusions

In conclusion, two methods for loading antimicrobials on PDA-coated glass slides were discussed: in situ loading of gentamicin followed by Ag NP immobilization on PDA coatings and silver and gentamicin loading on PDA-coated glass slides using the adsorption method. Their successful synthesis has been fully characterized by SEM, EDS and UV–visible spectrophotometry. Following this, further quantifications regarding the long-term release of loaded antimicrobials from PDA coatings were analyzed by ICP-AES and LC-MS. This uncovered the benefit of the proposed in situ loading method developed in this work, in regard to long-term slow release as compared to the post-loading method. In Ag@Gen/PDA coatings, the silver release rate was slower and long-term, which was attributed to the strong bonding with gentamicin and PDA as compared to Ag/Gen@PDA, where silver forms bonds with gentamicin and then adsorbs onto PDA. However, more silver was found loaded in Ag/Gen@PDA, which was attributed to silver reduction with the aid of gentamicin during loading as well as PDA. More silver loading makes the concentration gradient higher, which seemed to be the main driving force for silver release from loosely attached Ag/Gen@PDA, leading to faster silver release from such coatings. Therefore, it was concluded that the in situ loading method controls the silver release rate, ultimately producing long-term antimicrobial effects. Similar effects were observed with gentamicin release, which was quantified by LC-MS. These findings suggested in the in situ loaded method might be helpful for the long-term release of antimicrobials from as-prepared coatings.

In addition, there were various attempts to determine the synergistic antimicrobial properties of silver and gentamicin, viz., the zone of inhibition, optical density measurements and the spread plate method. The results were obvious according to combination-loaded antimicrobials when compared with single antimicrobial agents. Moreover, larger zones of inhibition and lower OD values were observed in Ag/Gen@PDA glass slides as compared to in situ loaded Ag@Gen/PDA coatings. This was clearly because of the faster release rate of loosely absorbed silver and gentamicin in Ag/Gen@PDA as compared to Ag@Gen/PDA-coated glass slides. As a result, the in situ loaded Ag@Gen/PDA glass coatings developed here might exhibit longer antimicrobial effects than post-loaded Ag/Gen@PDA glass coatings. Such antimicrobial coatings could thus be promising candidates to be implemented as biomedical implants. Further in vivo investigations will assist with such developments.

## Figures and Tables

**Table 1:** Percentage of Ag release in 30 days.

Samples	Total Ag (µg/cm^2^)	Ag Released in 30 Days (%)
Ag@PDA	10.4 ± 2.2	39.5 ± 1.4
Ag@Gen/PDA	10.3 ± 3.2	45.8 ± 1.6
Ag/Gen@PDA	26.4 ± 4.3	92.2 ± 0.9

**Table 2:** Results of zones of inhibition (ZOI).

Samples	ZOI against *S. aureus* (mm)	ZOI against *E. coli* (mm)
PDA	No ZOI formed	No ZOI formed
Ag@PDA	16.1 ± 1.1	14.6 ± 1.5
Gen/PDA	15.5 ± 1.5	15.9 ± 0.8
Ag@Gen/PDA	17.6 ± 0.8	16.8 ± 1.3
Ag/Gen@PDA	19.1 ± 1.0	17.8 ± 0.9

**Table 3:** Optical density (OD) values at 600 nm of nutrient broth incubated with glass slides.

Samples	OD_600_ against *S. aureus*	OD_600_ against *E. coli*
Glass	0.393 ± 0.002	0.767 ± 0.003
PDA	0.314 ± 0.017	0.754 ± 0.009
Ag@PDA	0.054 ± 0.007	0.067 ± 0.002
Gen/PDA	0.072 ± 0.021	0.072 ± 0.008
Ag/Gen@PDA	0.003 ± 0.001	0.003 ± 0.001
Ag@Gen/PDA	0.021 ± 0.002	0.014 ± 0.02

## Data Availability

The data that support the findings of this study are available from the corresponding author upon reasonable request.

## Supplementary Materials

The following supporting information can be downloaded at: https://www.mdpi.com/article/10.3390/molecules28114258/s1, Figure S1: (a) UV-visible spectra of PDA coated quartz slides at variable timings; and (b) Linear relationship of PDA coating with respect to polymerization time; Figure S2: Visual photographs obtained for PDA coatings; Figure S3: Possible mechanism of silver loading on PDA coated glass substrates; Figure S4: Duplicate petri dishes showing zones of inhibition against *S. aureus*. Numbers Petri dishes were numbered while an experiment; Figure S5: Duplicate petri dishes showing zones of inhibition against *E. coli*. Numbers Petri dishes were numbered while an experiment; Figure S6: 12-well plates showing the solution turbidity for each slide against (a) *S. aureus* and (b) *E. coli* respectively. Measurements were carried out in duplicates; and Figure S7: observation of bacterial growth on nutrient agar plates (a) *S. aureus* and (b) *E. coli*.

## Sample Availability

Samples of the compounds are not available from the authors.
