# Supplementary material for: Investigation of Antimicrobial Effects of Polydopamine-Based Composite Coatings
Source: Molecules. 2023 May 23;28(11):4258. doi: 10.3390/molecules28114258 (PMC10254676; doi:10.3390/molecules28114258)
Supplement: Supplementary file 1 [file molecules-28-04258-s001.zip › molecules-2385102-supplementary.pdf]

Investigation of Antimicrobial Effects of Polydopamine Based Composite Coatings

Rahila Batul[1,2], Mrinal Bhave[1] and Aimin Yu[1*]

[1] Department of Chemistry and Biotechnology, School of Science, Computing & Engineering Technologies, Swinburne University of Technology, Hawthorn, VIC 3122, Australia
[2] Department of Pharmacology and Toxicology, College of Pharmacy, University of Ha'il, City of Ha'il, 55211, Saudi Arabia

Supplementary Information

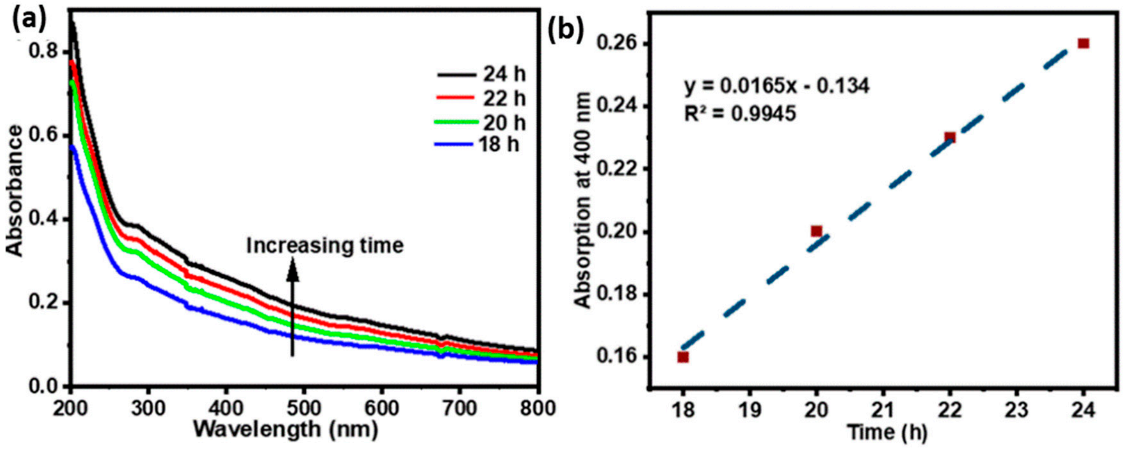

Figure S1: (a) UV-Visible spectra of PDA coated quartz slides at variable timings; and (b) Linear relationship of the absorbance of PDA coating with respect to polymerization time.

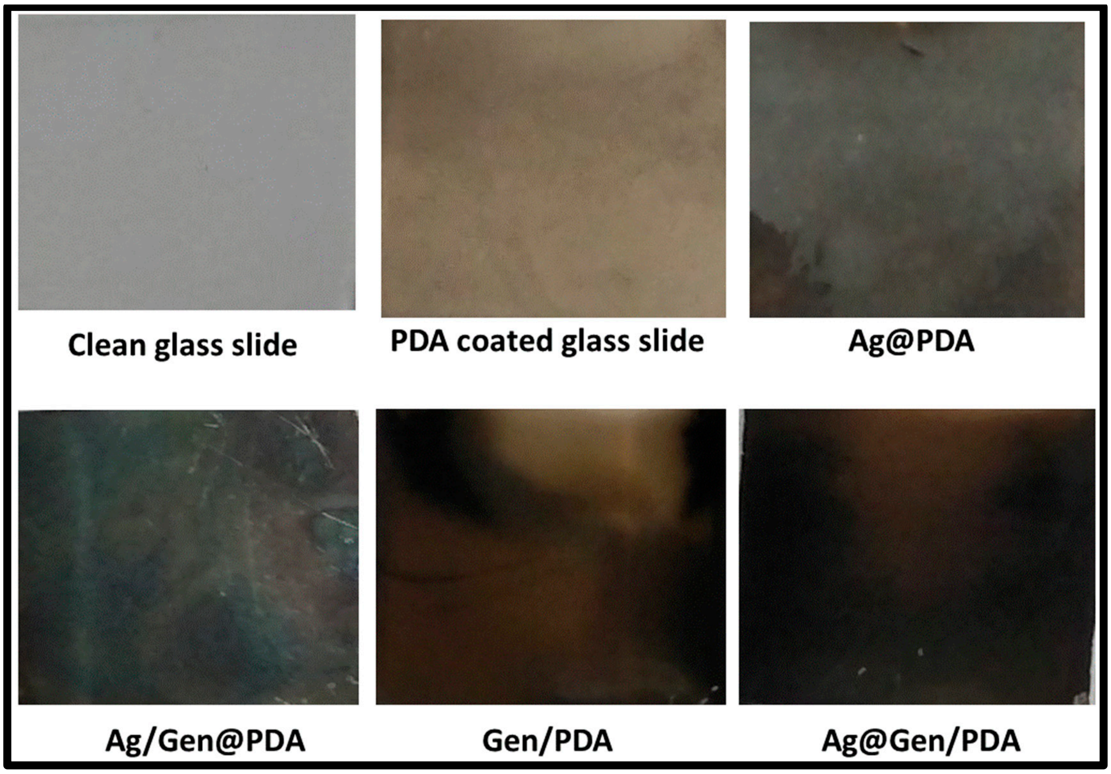

Figure S2: Visual photographs obtained for PDA coatings

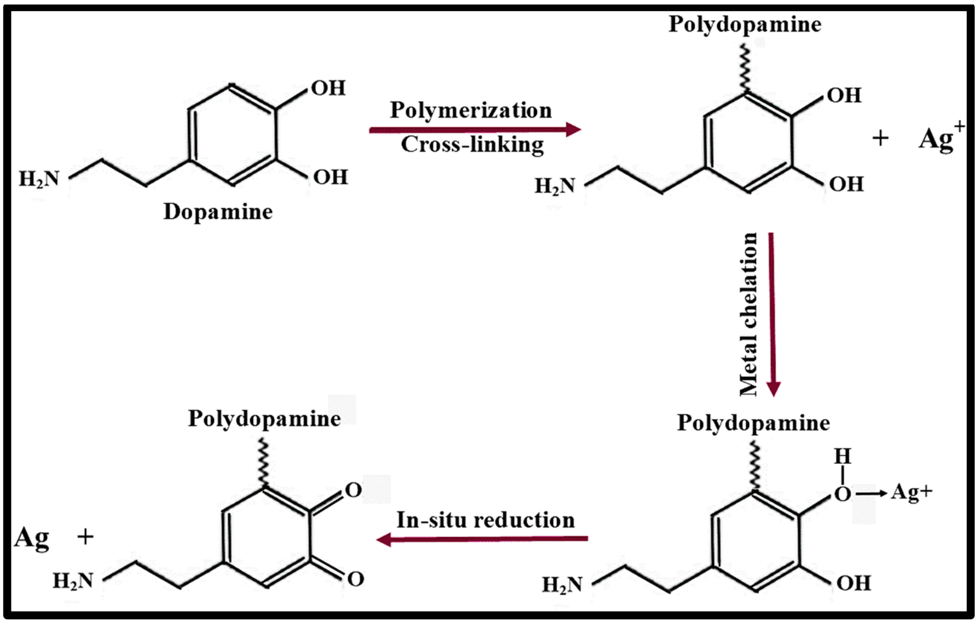

Figure S3: Possible mechanism of AgNPs loading on PDA coated glass substrates.

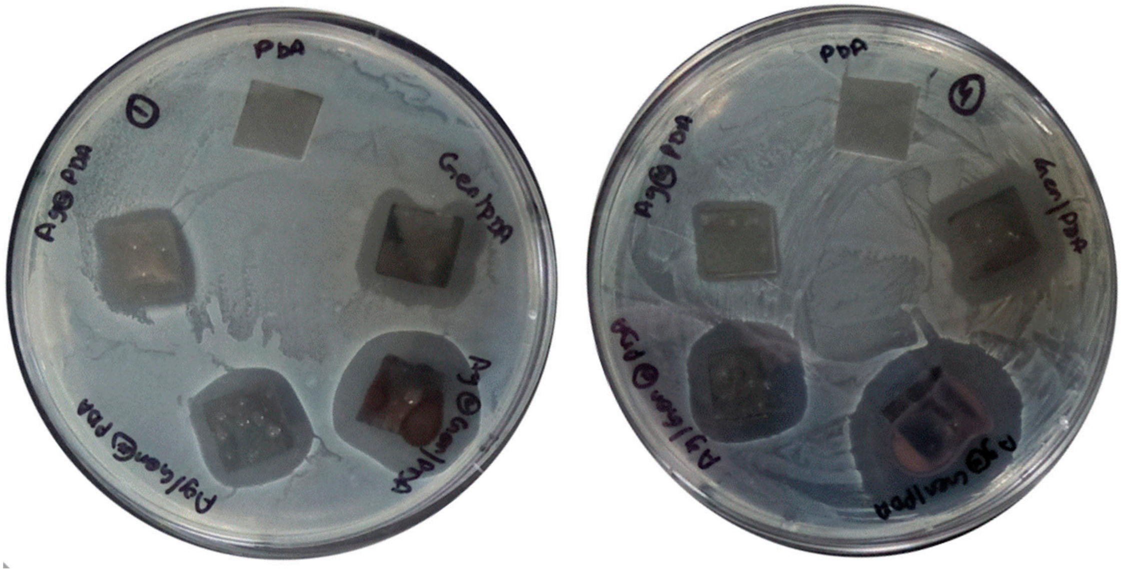

Figure S4: Duplicate petri dishes showing zones of inhibition against *S. aureus*. Numbers Petri dishes were numbered while an experiment.

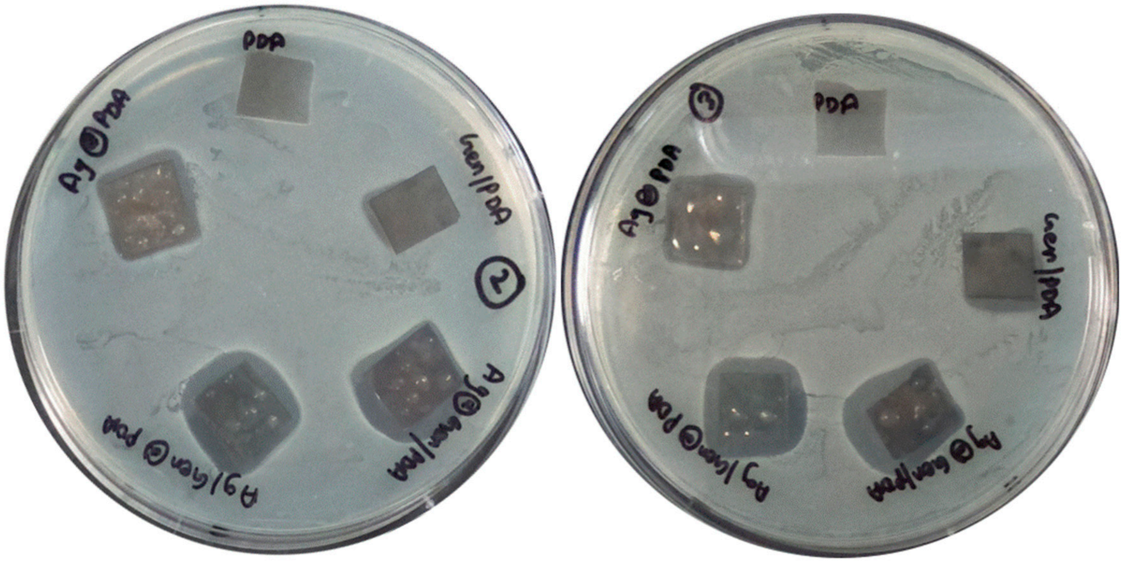

Figure S5: Duplicate petri dishes showing zones of inhibition against *E. coli*. Numbers Petri dishes were numbered while an experiment.

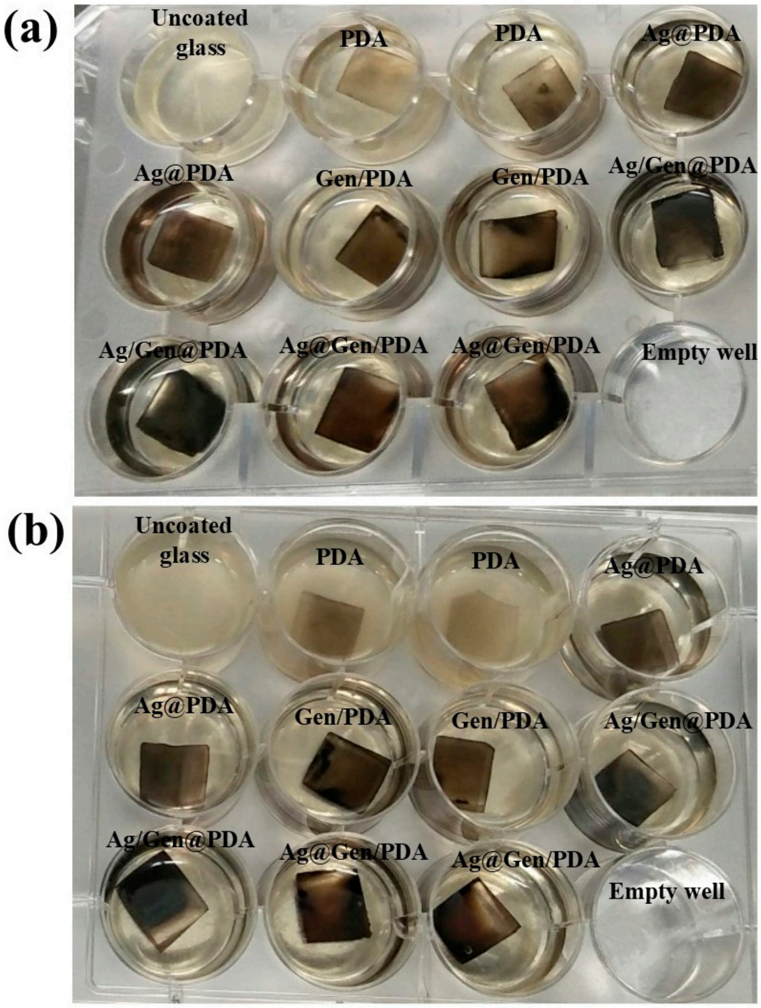

Figure S6: 12-well plates showing the solution turbidity for each slide against (a) *S. aureus* and (b) *E. coli* respectively. Measurements were carried out in duplicates.

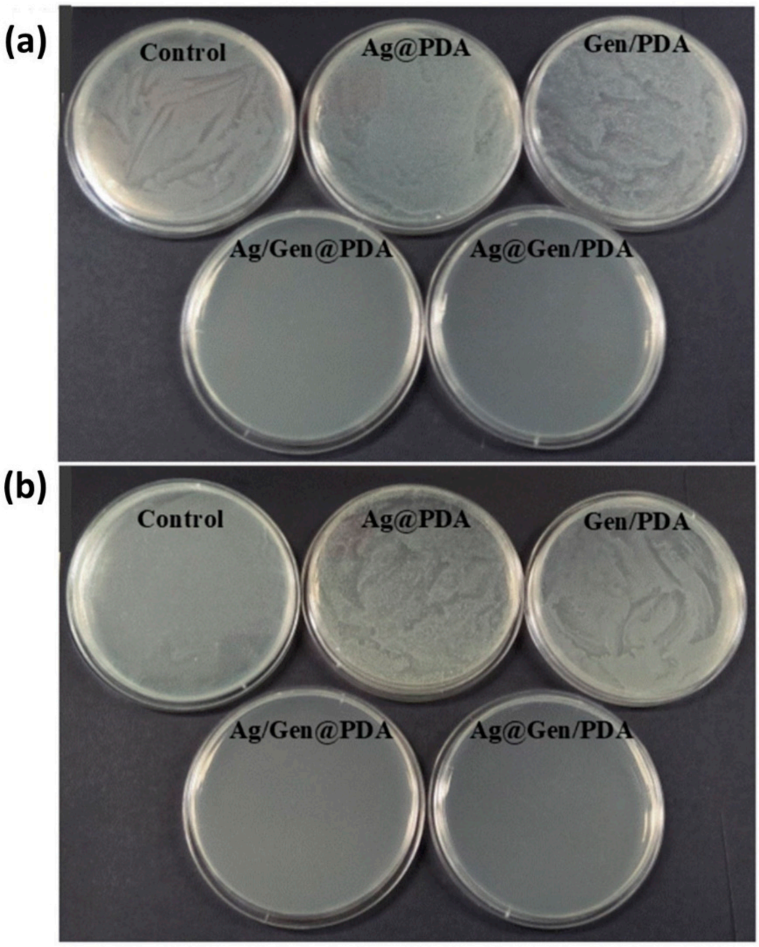

Figure S7: Observation of bacterial growth on nutrient agar plates against (a) *S. aureus* and (b) *E. coli*
